# Supplementary material for: Single-cell RNA sequencing reveals plasmid constrains bacterial population heterogeneity and identifies a non-conjugating subpopulation
Source: Nat Commun. 2024 Jul 12;15:5853. doi: 10.1038/s41467-024-49793-x (PMC11245611; doi:10.1038/s41467-024-49793-x)
Supplement: Supplementary file 1 — Supplementary Information [file 41467_2024_49793_MOESM1_ESM.pdf]

Single-cell RNA sequencing reveals plasmid constrains bacterial population heterogeneity and identifies a non-conjugating subpopulation

Valentine Cyriaque, Rodrigo Ibarra-Chávez, Anna Kuchina, Georg Seelig, Joseph Nesme, Jonas Stenløkke Madsen

Supplementary tables and figures

Tab.S1. Growth parameters of P. putida KT2440 (WT) and *P. putida* KT2440/pKJK5 when grown separately over 24 hours. Growth curve is displayed in figure 1. Detailed statistics can be found in tab.S4

	**Growth rate (*r*)**	**Area Under the Curve**
P. putida KT2440/pKJK5	0.78	23.43
	0.75	23.44
	0.79	23.60
	0.80	23.14
	0.79	23.10
	0.74	23.89
	0.77	23.31
	0.75	23.19
	0.73	24.06
	0.72	23.57
	0.75	23.31
P. putida KT2440 WT	1.23	25.54
	1.09	25.07
	1.16	24.86
	1.16	25.00
	1.33	24.22
	1.15	24.46
	1.11	24.96
	1.20	24.48
	1.09	24.91
	1.17	25.18
	1.03	24.94
	1.06	25.41
statistical parameters	Welch Two Sample t-test (two-sided)<br>t =-15.575 ;<br>p= 5.12E-10	Student's t-test (two-sided)<br>t = -10.045 ;<br>p=1.792E-09

Tab.S2. Plasmid fitness cost obtained from a competition experiment between *Pseudomonas putida* KT2440 and *Pseudomonas putida* KT2440/pKJK5 in a liquid overnight agitated (250RPM) co-culture to avoid plasmid transfer between plasmid free and carrier cells.

	CFU counts						
	Before competition assay			After competition assay			
replicate	LB (total CFU)	tetracyclin ($Pcarrier_i$)	CFU difference ($Pfree_i$)	LB (total CFU)	tetracyclin ($Pcarrier_f$)	CFU difference ($Pfree_f$)	w=ln($Pcarrier_f$/$Pcarrier_i$) / ln($Pfree_f$/$Pfree_i$)
1	6460	1470	4990	65800000	37900000	27900000	1,018
2	7100	1530	5570	106600000	60300000	46300000	1,015
3	6070	3250	2820	106300000	64900000	41400000	1,026
4	7030	3030	4000	107700000	55700000	52000000	1,004

Tab.S3. Average transcripts/cell for plasmid free (P-) and carrier (P+) cells at OD 0.5 and 1.5 in the main experiment (E2) after removing cells with less than 85 transcripts and features absent in more than 10% of cells from each growth condition.

OD	Plasmid	Cells	Mean gene count ± SD	Mean transcript count ± SD
0.5	P-	499	168.80±81.12	371.63±311.72
1.5	P-	366	169.13±87.32	398.51±381.96
0.5	P+	206	181.17±83.69	398.29±292.48
1.5	P+	415	171.67±82.78	349.27±271.34

Tab.S4. Average transcripts/cell for clusters defined with the whole transcriptome (plasmid and chromosome encoded genes), after removing cells with less than 85 transcripts and features absent in more than 10% of cells from each growth condition.

W cluster	Cells	Mean gene count ± SD	Mean transcript count ± SD
W1	143	239.63±62.60	577.44±277.80
W2	86	101.34±22.71	161.31±53.07
W3	177	124.55±26.23	216.89±65.48
W4	170	250.86±67.34	643.14±356.91
W5	66	145.62±60.11	293.56±174.80
W6	194	90.94±14.39	131.39±26.76
W7	145	264.79±63.02	627.84±282.16
W8	128	145.33±20.56	253.02±54.47
W9	175	97.99±18.18	155.83±43.27
W10	142	184.65±26.80	388.16±102.38
W11	60	323.38±65.87	1057.43±505.45

Tab.S5. Average transcripts/cell for clusters defined with the chromosome-encoded gene transcriptome defined clusters (chromosome encoded genes), after removing cells with less than 85 transcripts and features absent in more than 10% of cells from each growth condition.

Ch cluster	Cells	Mean gene count ± SD	Mean transcript count ± SD
**Ch1**	248	109.31±26.13	182.01±65.29
**Ch2**	168	224.00±68.33	539.71±295.12
**Ch3**	167	237.34±57.75	583.34±279.17
**Ch4**	66	143.24±59.30	287.36±172.95
**Ch5**	163	87.41±11.73	126.75±23.62
**Ch6**	210	282.96±72.30	772.35±441.02
**Ch7**	174	140.03±23.19	244.96±65.29
**Ch8**	146	99.12±18.16	159.82±44.27
**Ch9**	135	185.70±26.51	390.72±102.70

Tab.S6. Parameters associated with gene transcript network (Fig. S13) obtained from Spearman correlation between genes amongst cells transcripts from the same growth condition (P-0.5, P+0.5, P-1.5, P+1.5) after removing cells with less than 85 transcripts and subsampling the dataset to 206 cells with the highest number of transcripts.

**subsampled**	**P- OD0.5**	**P+ OD0.5**	**P- OD1.5**	**P+ OD1.5**
Nodes	201	252	219	246
Edges	696	689	1001	1105
Avg number of neighbors	7.203	5.668	9.441	9.252
Network diameter	8	9	8	8
Network radius	5	5	4	4
Characteristic path length	3.176	3.527	2.984	3.001
Clustering coefficient	0.104	0.044	0.114	0.130
Network density	0.038	0.024	0.45	0.039
Network heterogeneity	0.986	0.915	0.880	0.923
Network centralization	0.154	0.090	0.181	0.169
Connected components	6	6	4	5
**Average correlation**	0.657	0.655	0.657	0.657
**Cells**	206	206	206	206

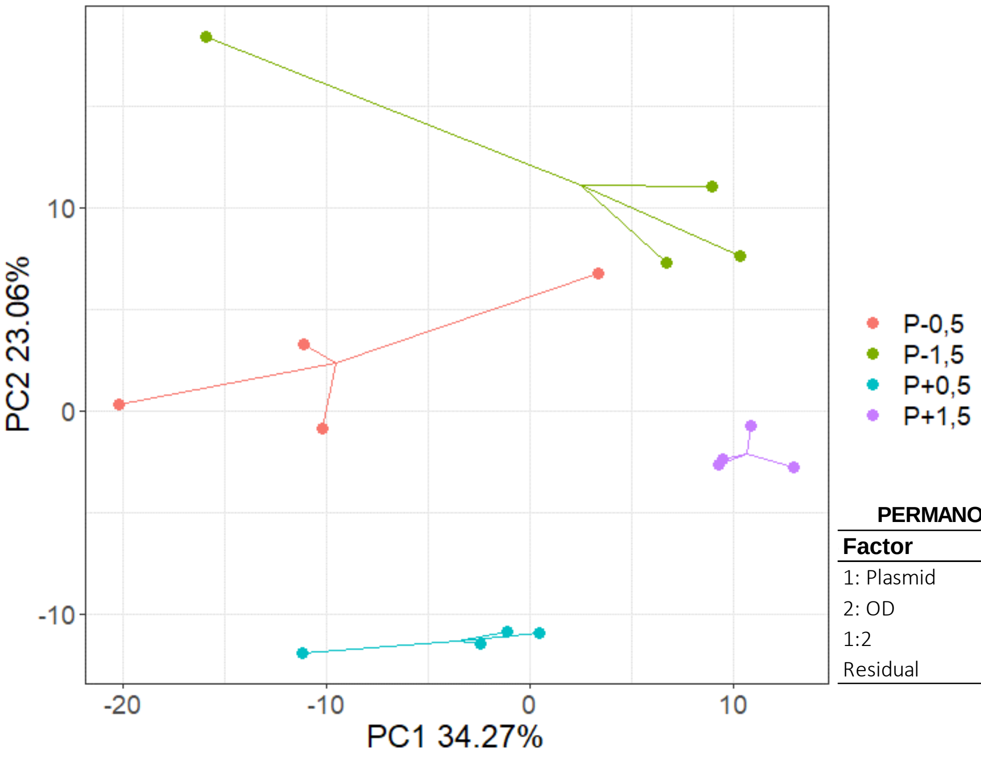

PERMANOVA (Euclidean dist., 10,000 permutations)

**Factor**	***r²***	***p***	**Signif.**
1: Plasmid	0.141	0.0173	*
2: OD	0.246	0.0006	**
1:2	0.026	0.8540	-
Residual	0.58652	-	-

Fig.S1. PCA multivariate analysis separated population level (bulk) transcriptomes (plasmid and chromosome encoded genes) according to ODs and the presence of the plasmid. as confirmed using 10,000 permutations in a Permutational multivariate analysis of variance (n= 4 independent experiments).

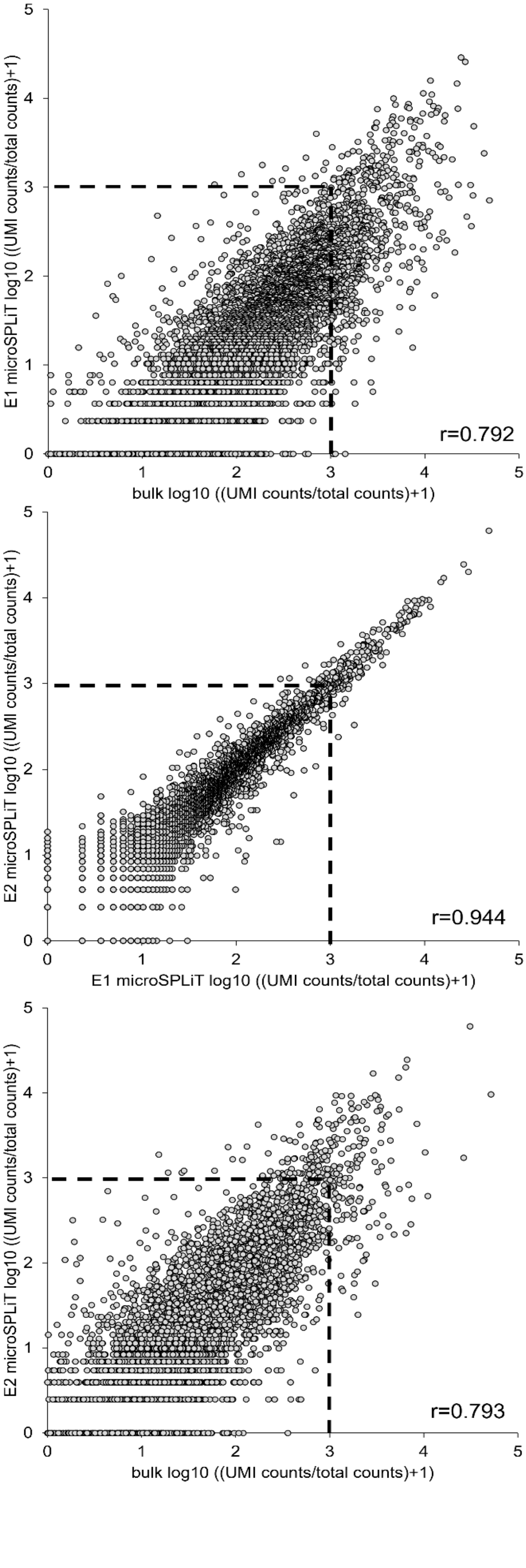

Fig.S2. Correlation coefficient calculated between the summed single-cell expressions per gene data of microSPLiT experimental replicates (identified here as E1 - blind control experiment or E2, microSPLiT experiment : n:2 indepentant experiments) or between the summed single-cell expressions per gene data from one of the microSPLiT replicates and the bulk population level (n=4 independent bulk transcriptomes).

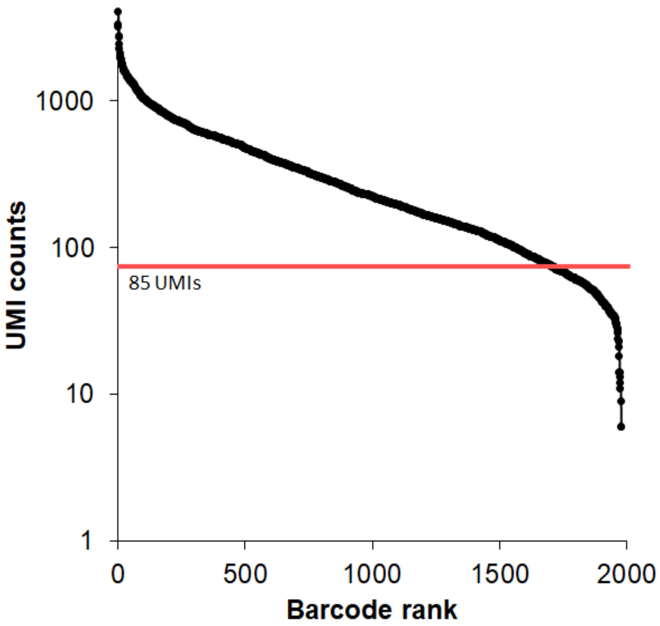

Fig.S3. UMI counts distribution from high to low count depths in microSPLiT sc-RNA sequencing experimental replicates (E2). Selected cells for the following analyses counted more than 85 UMIs (red line) to avoid cell count bias in cell clustering.

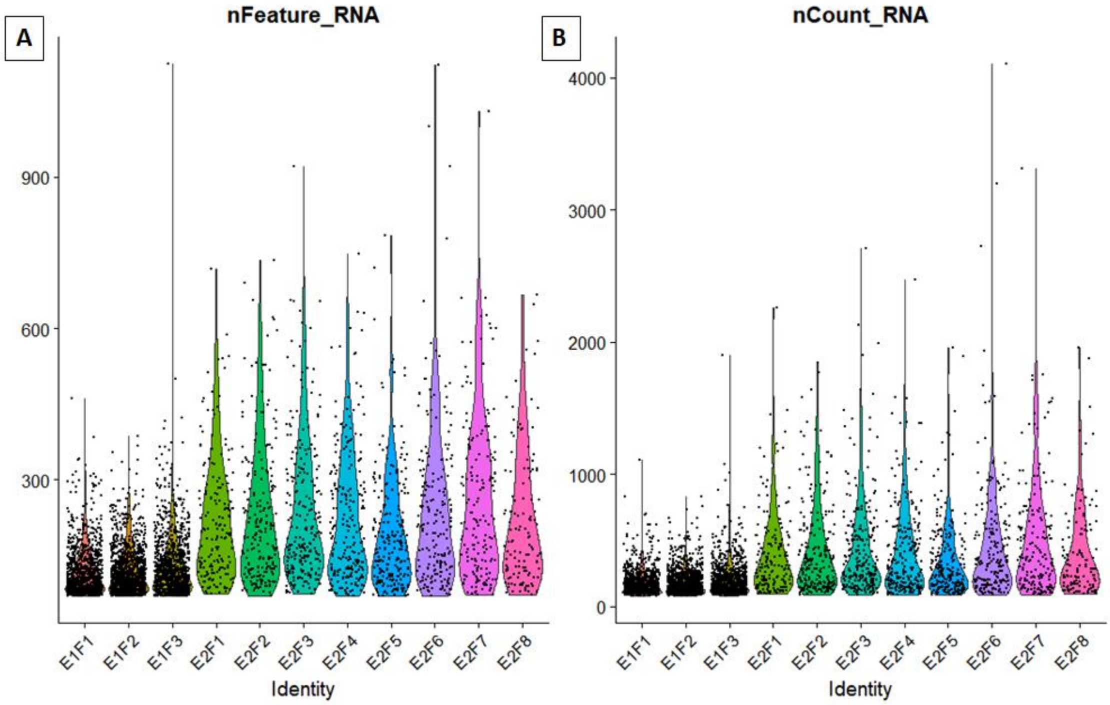

Fig.S4. Amount of gene features per cell (A) or UMI count per cell (B) among the microSPLiT sc-RNA sequencing blind control experiment (E1) and microSPLiT sc-RNA sequencing experimental replicates (E2) in the sub-libraries (3 sublibrairies in E1, 8 sublibraires in E2). Sublibraries prepared during the microSPLiT sample preparation displayed an equivalent number of transcripts (nCounts) and genes (nFeatures). This was maintained after filtering out genes absent from more than 10% of each cell growth condition groups (Fig.S5).

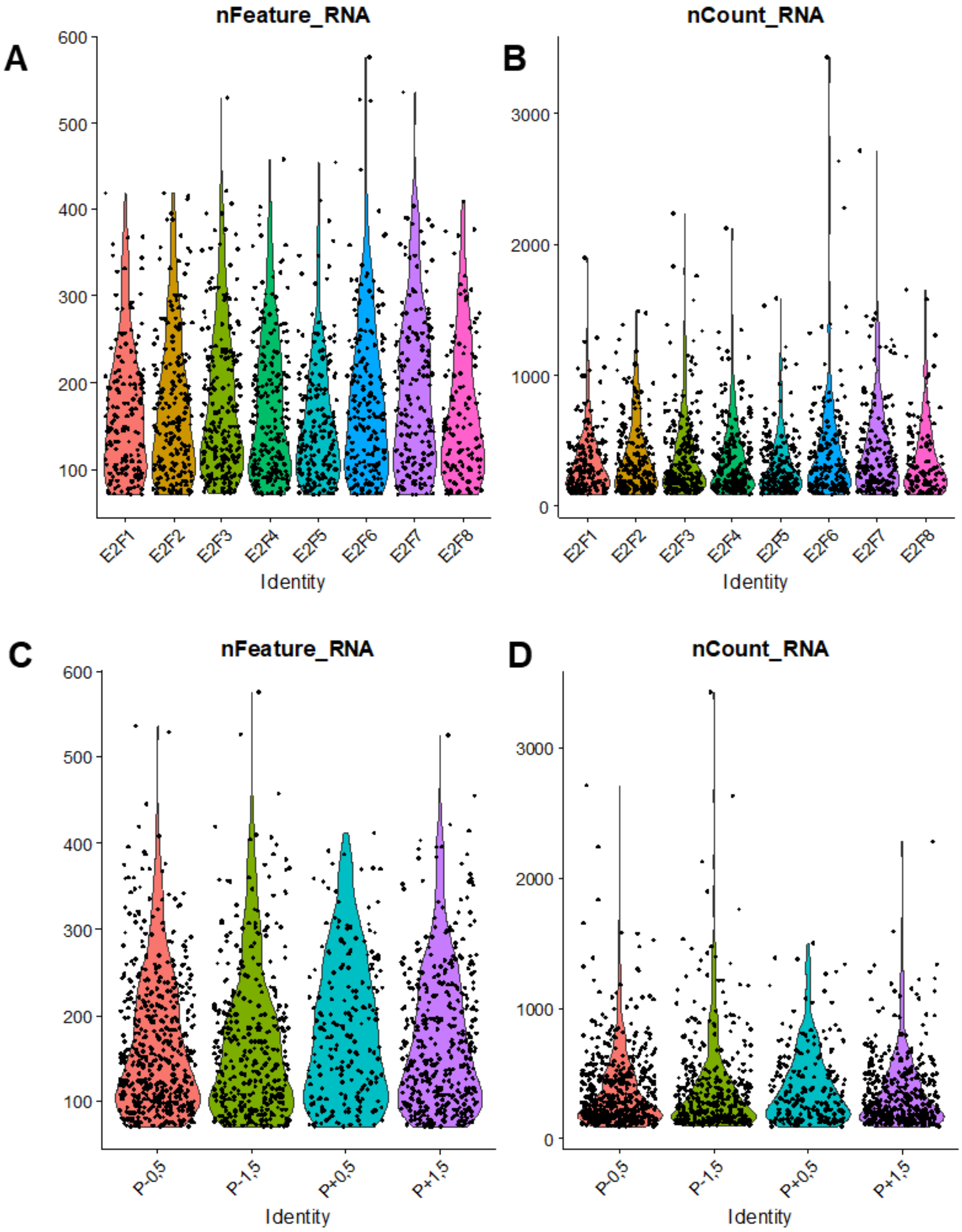

Fig.S5. Amount of gene features per cell (A, C) or UMI count per cell (B, D) among the microSPLiT sc-RNA sequencing experimental replicates (E2) in the 8 F sub-libraries (A-B) or in cell categories (P-0.5, P-1.5, P+0.5, P+1.5 ; C-D) after filtering out genes present in less than 10% of cells in each category.

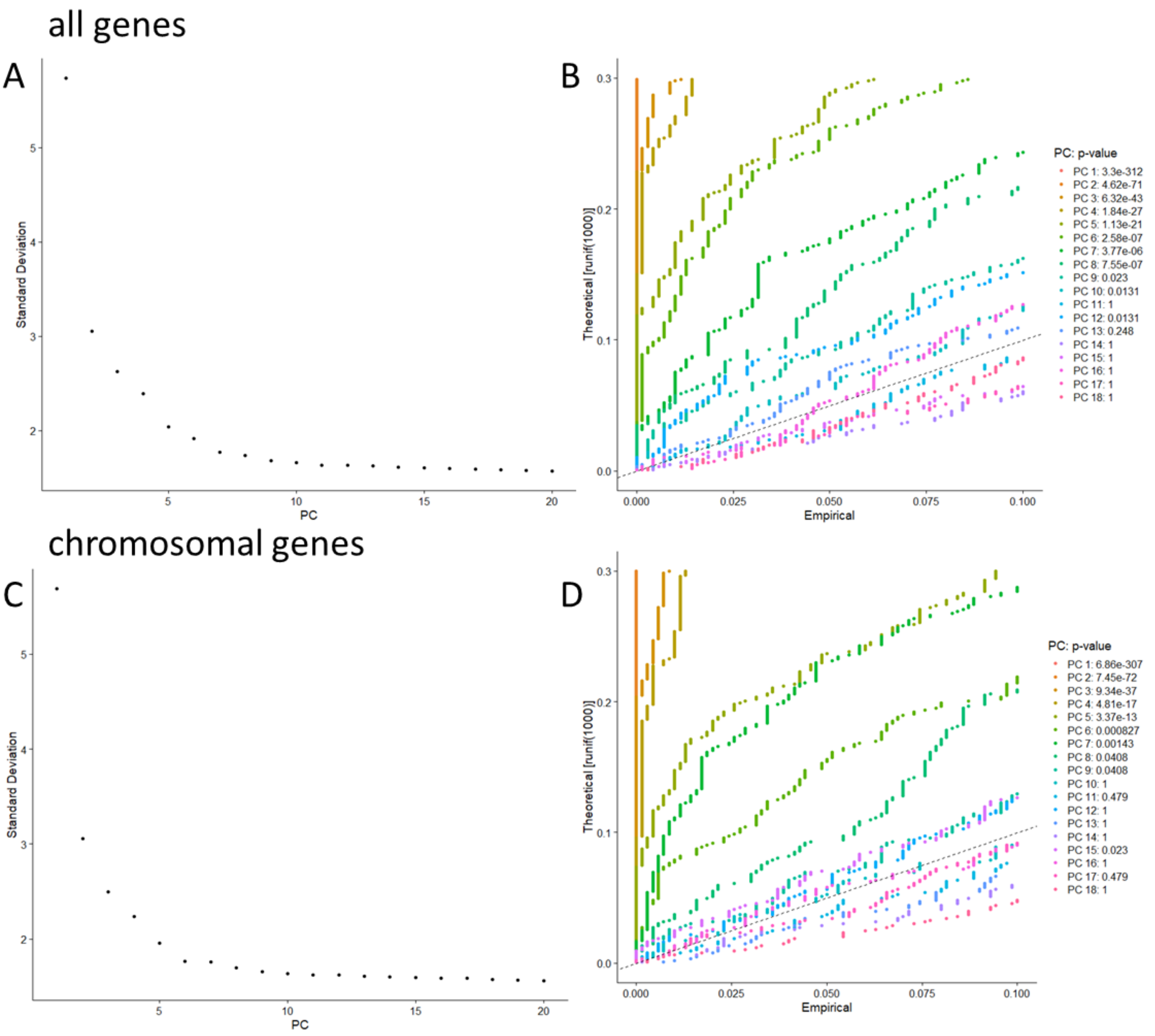

Figures S6: Principal components' standard deviations (A,C) and JackStraw plots after 100 resampling (B,D) obtained from PCAs carried for all-genes and chromosome-encoded genes dataset after filtering out genes displaying transcripts in less than 10 % of the cells from all growth conditions (P-0.5. P-1.5. P+0.5. P+1.5). Test and figures were generated using the *Seurat* R package.

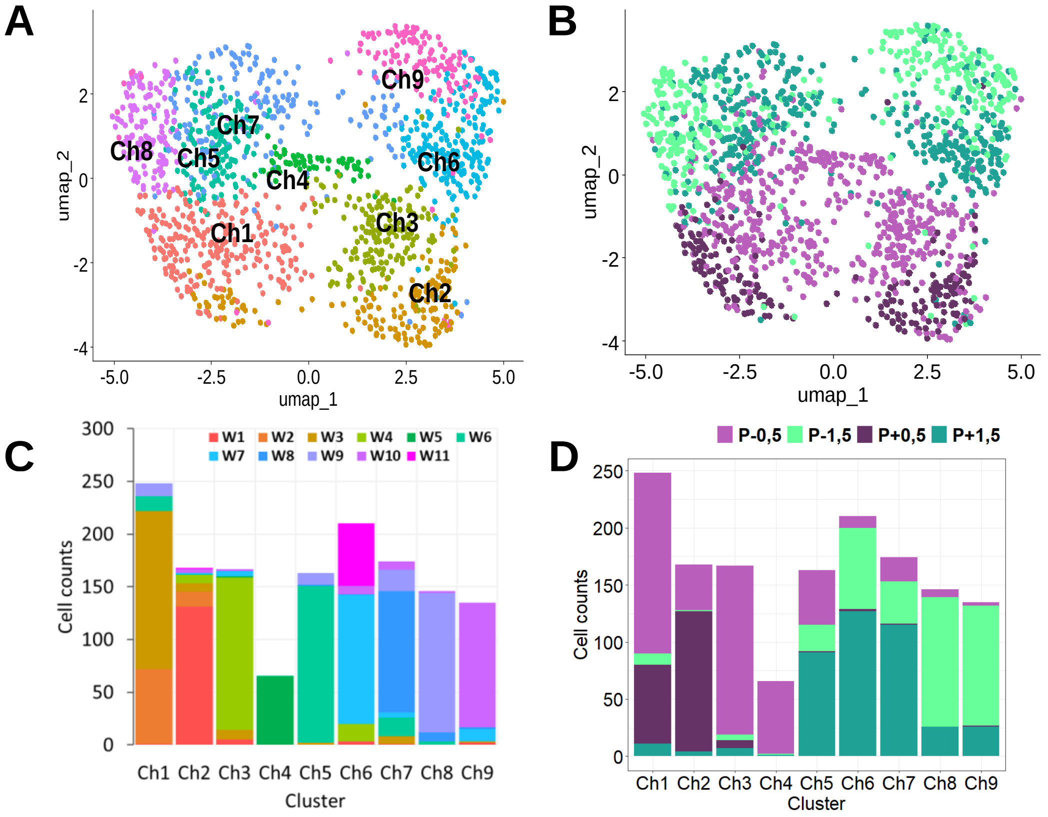

Fig. S7. Subpopulation clustering identified by single-cell transcriptomics of *P. putida* carrying a plasmid (P+) or not (P-) at early (OD0.5) and late exponential growth (OD1.5) using chromosome encoded-genes only (n= 1 independent experiment ; n= 1477 cells with >85 transcripts)). (A) UMAP obtained by microSPLiT scRNA sequencing identified 9 chromosome-encoded transcriptome clusters (Ch1-Ch9). Cell count in each cluster can be found in Tab. S5. (B) Clusters segregate cell transcriptomes in subpopulations separating the two different growth states (OD0.5 and 1.5). (C) W cluster membership of cells from clusters C1-9 (D) Cell transcriptomes from OD0.5 were mainly distributed in clusters Ch1-4, while cells from OD1.5 were mainly distributed in clusters Ch5-9.

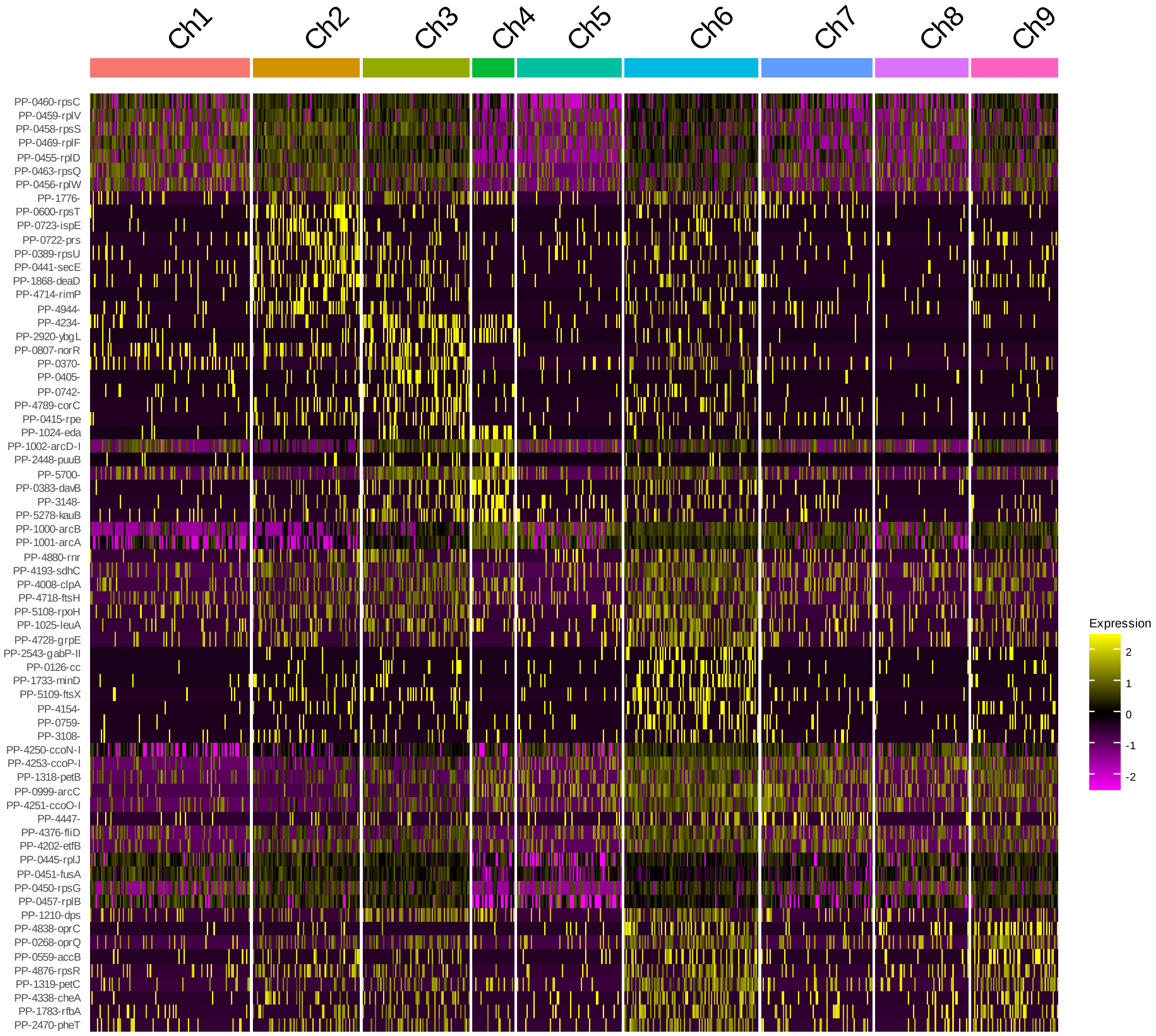

Fig.S8. Heatmap of transcription level displaying the top 10 bio-marker genes (Wilcoxon signed-rank test, (p<0.05)) of Ch1-7 clusters identified with FindAllMarkers from the *Seurat* R package. Genes appearing in the top-8 of several clusters are displayed only one time. Colours represent normalized centralized transcript abundance.

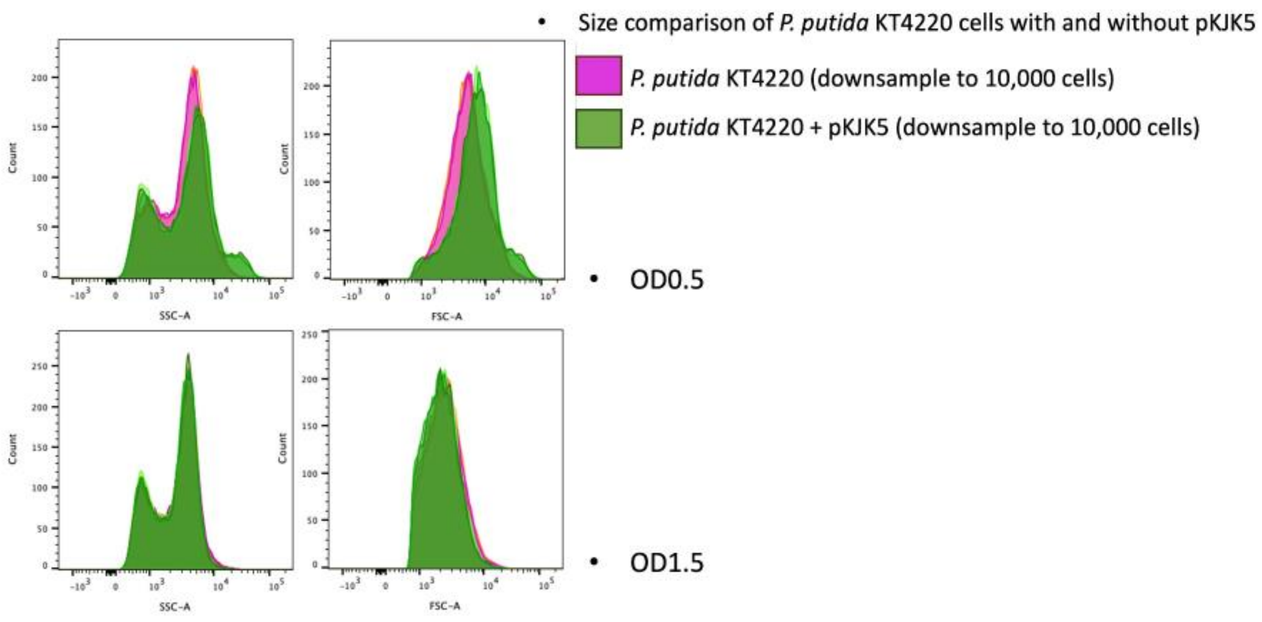

Fig.S9. Cell size and granulometry of plasmid free (*Pseudomonas putida* KT2440) and carrier (*Pseudomonas putida* KT2440/pKJK5) cells evaluated through flow-cytometry (SSC (left) and FSC (right) channels respectively. exc. 488 nm) . Experiment was repeated 3 times with similar results.

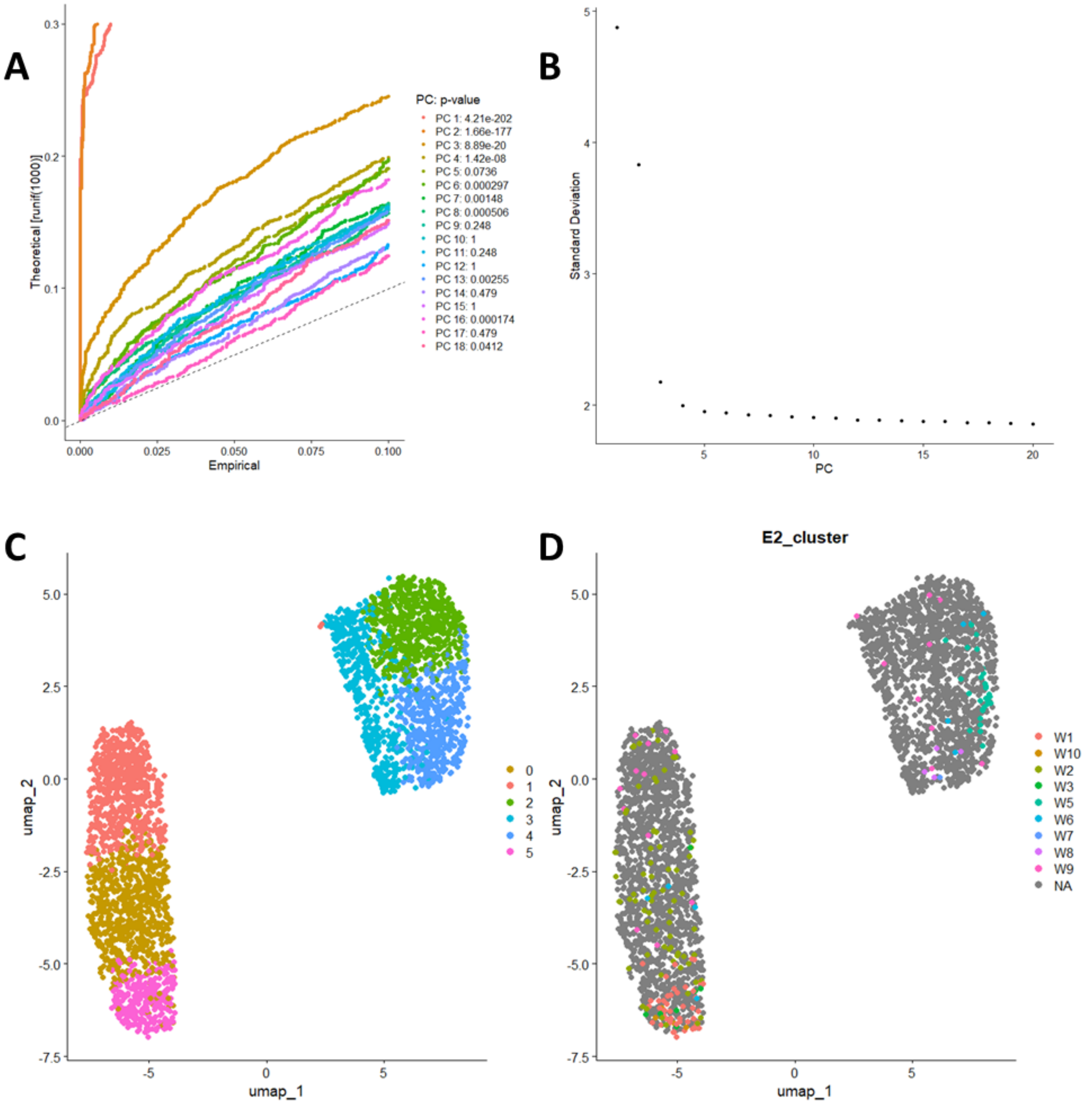

Fig.S10. Blind control microSPLiT experiment characterization. Principal components' standard deviations (A) and JackStraw plots after 100 resampling (B) obtained from PCAs carried for chromosome-encoded genes datasets E1 (control) and E2 (experiment) after filtering out E2 cells forming its own cluster (n= 2 independent experiments). (C-D) UMAP (8 dimensions) of transcriptomes of *Pseudomonas putida* obtained with microSPLiT sc-RNA sequencing (control E1 and experiment E2) (C) identifying 6 clusters and where (D) E2 cells' W cluster identity were be displayed (W1-10). Grey dots represent cells from E1 experiment.

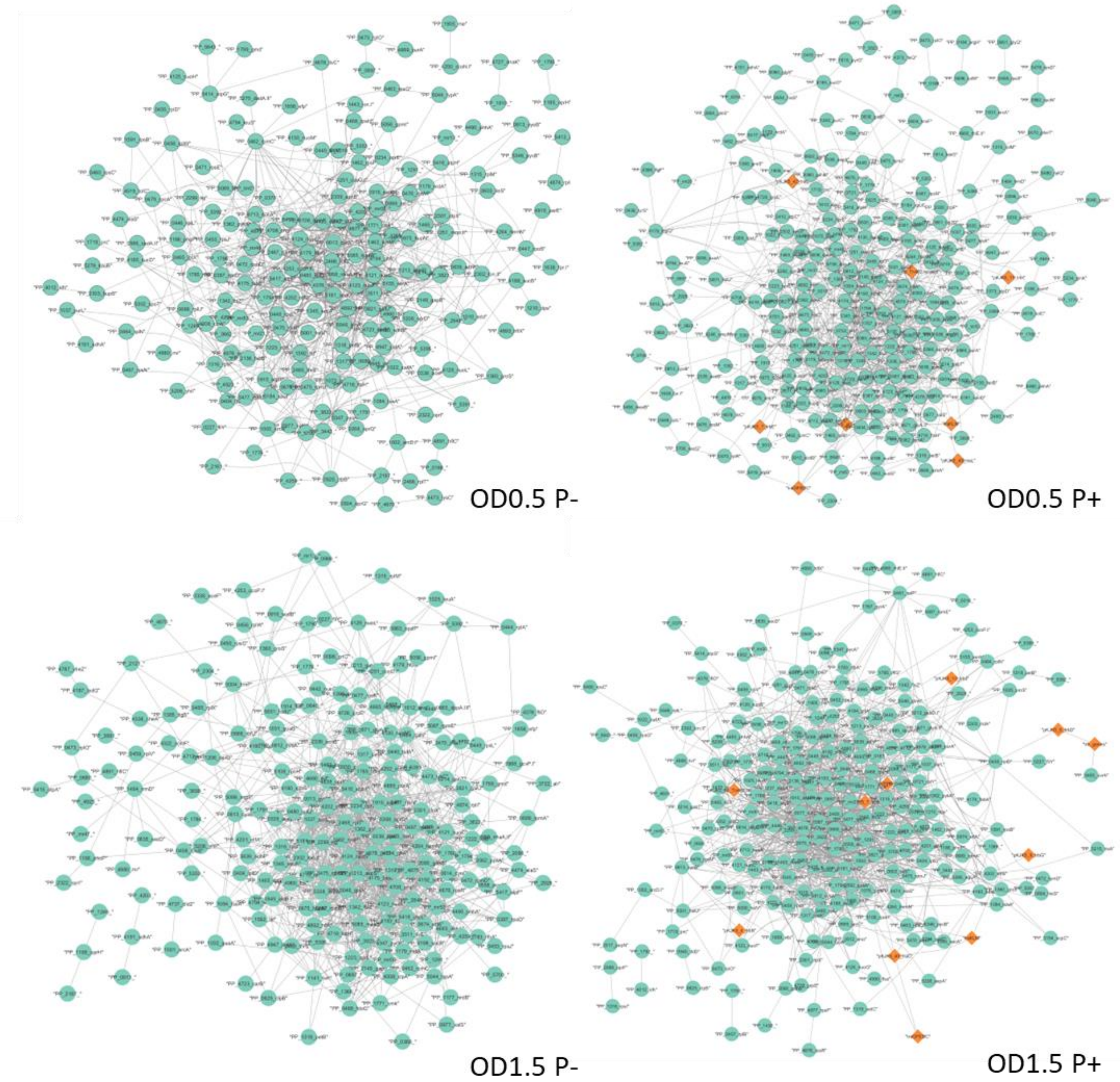

Fig.S11. Gene transcript network (Fig. S13) obtained from Spearman correlation between genes amongst cells transcripts from the same growth condition (P-0.5, P+0.5, P-1.5, P+1.5) after removing cells with less than 85 transcripts and subsampling the dataset to n=206 cells (= minimum number of cells per group) . Each dot correspond to 1 gene transcript (blue for chromosomal transcript and orange for plasmid-encoded transcript) and lines represent positive ($rs>0.6$, $p<0.5$, $n>30$) between those. Cell transcriptomes were colored according to the presence of plasmid encoded transcripts in their transcriptomic profile. In plasmid free cells (red). no plasmid encoded transcript was identified ; in plasmid carriers (blue)). at least 1 plasmid transcript was identified.

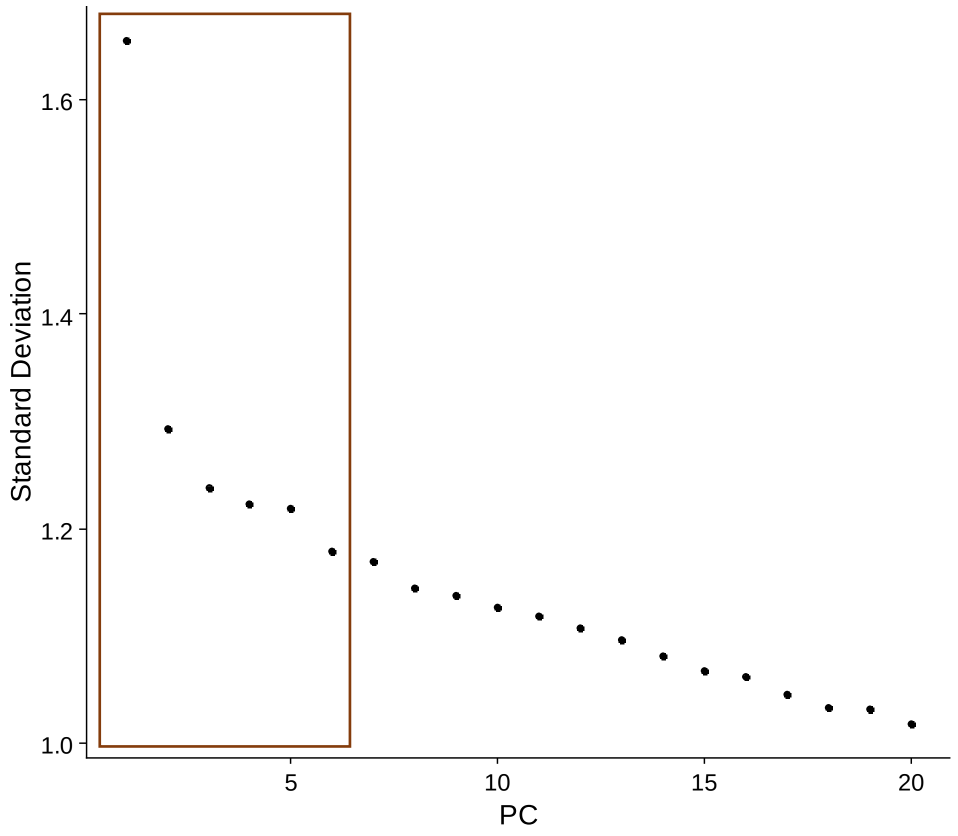

Figure S12. Principal components' standard deviations obtained from PCAs carried for chromosome-encoded genes datasets E2 using plasmid genes only. The 9 first dimensions were used for finding cell clustering and UMAP projection (Fig. S14).

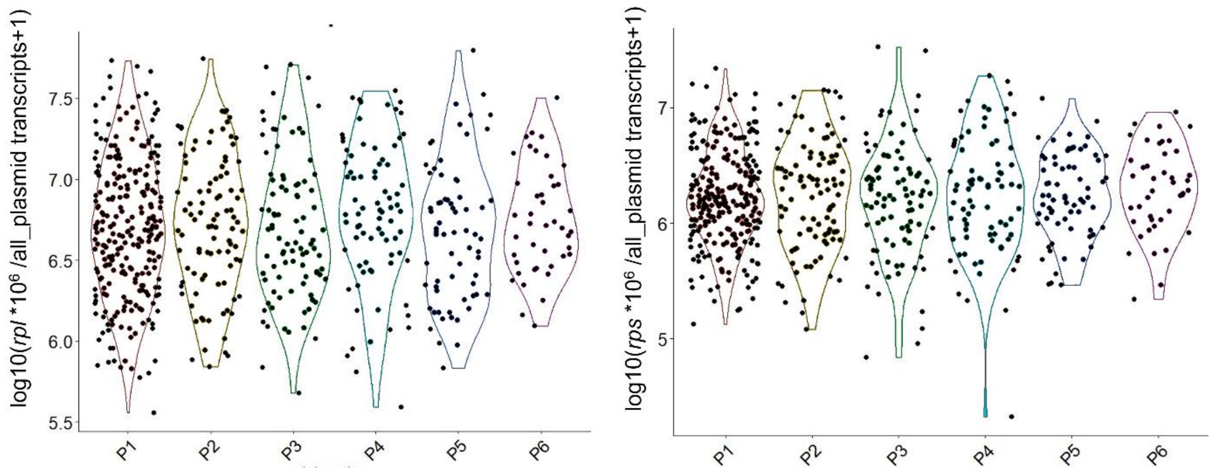

Figure S13. Normalized summed transcript counts of *rpl* and *rps* genes genes from individual cells displayed on top of the UMAP generated with the plasmid single-cell transcriptomes and on a violin plot according to P clusters.
