# Supplementary material for: Single-cell RNA sequencing reveals plasmid constrains bacterial population heterogeneity and identifies a non-conjugating subpopulation
Source: Nat Commun. 2024 Jul 12;15:5853. doi: 10.1038/s41467-024-49793-x (PMC11245611; doi:10.1038/s41467-024-49793-x)
Supplement: Supplementary file 3 — Description of Additional Supplementary Files [file 41467_2024_49793_MOESM3_ESM.pdf]

Description of Additional Supplementary Files:

Supplementary Data 1: Top 30 marker genes (p-value <0.05) defining W clusters and associated p-value (Wilcoxon Rank Sum test). If clusters have less than 30 marker genes, only the significant ones will be displayed in this table.
