# Supplementary material for: Single-cell RNA sequencing reveals plasmid constrains bacterial population heterogeneity and identifies a non-conjugating subpopulation
Source: Nat Commun. 2024 Jul 12;15:5853. doi: 10.1038/s41467-024-49793-x (PMC11245611; doi:10.1038/s41467-024-49793-x)
Supplement: Supplementary file 5 — Reporting Summary [file 41467_2024_49793_MOESM5_ESM.pdf]

nature portfolio

Corresponding author(s): Valentine Cyriaque, Jonas S. Madsen

Last updated by author(s): Oct 11, 2023

Reporting Summary

Nature Portfolio wishes to improve the reproducibility of the work that we publish. This form provides structure for consistency and transparency in reporting. For further information on Nature Portfolio policies, see our Editorial Policies and the Editorial Policy Checklist.

Statistics

For all statistical analyses, confirm that the following items are present in the figure legend, table legend, main text, or Methods section.

n/a	Confirmed	
☐	☒	The exact sample size (*n*) for each experimental group/condition, given as a discrete number and unit of measurement
☐	☒	A statement on whether measurements were taken from distinct samples or whether the same sample was measured repeatedly
☐	☒	The statistical test(s) used AND whether they are one- or two-sided<br>*Only common tests should be described solely by name; describe more complex techniques in the Methods section.*
☒	☐	A description of all covariates tested
☐	☒	A description of any assumptions or corrections, such as tests of normality and adjustment for multiple comparisons
☐	☒	A full description of the statistical parameters including central tendency (e.g. means) or other basic estimates (e.g. regression coefficient) AND variation (e.g. standard deviation) or associated estimates of uncertainty (e.g. confidence intervals)
☐	☒	For null hypothesis testing, the test statistic (e.g. *F*, *t*, *r*) with confidence intervals, effect sizes, degrees of freedom and *P* value noted<br>*Give P values as exact values whenever suitable.*
☒	☐	For Bayesian analysis, information on the choice of priors and Markov chain Monte Carlo settings
☒	☐	For hierarchical and complex designs, identification of the appropriate level for tests and full reporting of outcomes
☒	☐	Estimates of effect sizes (e.g. Cohen's *d*, Pearson's *r*), indicating how they were calculated

Our web collection on statistics for biologists contains articles on many of the points above.

Software and code

Policy information about availability of computer code

Data collection	The sequence data generated in this study have been deposited in the National Center for Biotechnology Information's SRA repository under accession code PRJNA1019643 [https://www.ncbi.nlm.nih.gov/bioproject/PRJNA1019643/]. The reference genome data used in this study were generated combining the pKJK5 plasmid sequence available in the NCBI database under accession code AM261282.1 [https://www.ncbi.nlm.nih.gov/nuccore/AM261282] and Pseudomonas putida KT2440 genome available in the Ensembl Bacteria database under accession code GCA_000007565 [https://bacteria.ensembl.org/Pseudomonas_putida_kt2440_gca_000007565]. The mapping, demultiplexing and quantification for bulk and single cell RNA-seq data generated in this study have been deposited in the Zenodo database under accession code 11356666 [https://doi.org/10.5281/zenodo.11356666].
Data analysis	Statistical analyses were performed with R 4.3.2 (packages vegan-2.6-4, mvabund-4.2.1, Seurat-5.0.1, patchwork-1.2.0, dplyr-2.4.0, scCustomize-2.0.1, ggplot2-3.4.4, rcorr -5.1-2 and corrplot-0.92). The sequenced reads were trimmed of the remaining adapter sequences and low quality based using bbduk (BBMap 38-90-Bushnell B. - sourceforge.net/projects/bbmap/). Reads were mapped against the reference genome, and the per-cell gene counts were quantified using STAR-2.7.9a (bulk trascriptomics) or STARsolo from STAR-2.7.9a (single-cell transcriptomics) (https://github.com/alexdobin/STAR). Flow Cytometry analyses were obtained with FlowJo software v10 (Tree Star Inc., USA).

For manuscripts utilizing custom algorithms or software that are central to the research but not yet described in published literature, software must be made available to editors and reviewers. We strongly encourage code deposition in a community repository (e.g. GitHub). See the Nature Portfolio guidelines for submitting code & software for further information.

Data

Policy information about availability of data

All manuscripts must include a data availability statement. This statement should provide the following information, where applicable:

- Accession codes, unique identifiers, or web links for publicly available datasets
- A description of any restrictions on data availability
- For clinical datasets or third party data, please ensure that the statement adheres to our policy

Data collection from public repositories has been aknowledged as pKJK5 plasmid GenBank record AM261282.1 and Pseudomonas putida KT2440 genome (ASM756v2 on EnsemblBacteria).
All sequence data produced in this publication are publicly available through the National Center for Biotechnology Information's SRA repository under accession number PRJNA1019643.

Research involving human participants, their data, or biological material

Policy information about studies with human participants or human data. See also policy information about sex, gender (identity/presentation), and sexual orientation and race, ethnicity and racism.

Reporting on sex and gender	Not applicable
Reporting on race, ethnicity, or other socially relevant groupings	Not applicable
Population characteristics	Not applicable
Recruitment	Not applicable
Ethics oversight	Not applicable

Note that full information on the approval of the study protocol must also be provided in the manuscript.

Field-specific reporting

Please select the one below that is the best fit for your research. If you are not sure, read the appropriate sections before making your selection.

☒ Life sciences ☐ Behavioural & social sciences ☐ Ecological, evolutionary & environmental sciences

For a reference copy of the document with all sections, see nature.com/documents/nr-reporting-summary-flat.pdf

Life sciences study design

All studies must disclose on these points even when the disclosure is negative.

Sample size	For single-cell transcriptomics, cells with less than 85 UMI counts were sorted out. This treshold was chosen to be above the sharp drop of UMIs as determined with the knee-graph (UMI count per barcode rank [51]; figure S3). This left samples sizes of 3165 cells (Control E1 experiment) and 1486 cells (E2 experiment). We consider that this sample size was sufficient since cell identity could be retraced from the clustering [51].
Data exclusions	No data exclusion
Replication	Bulk transcriptomics, flow cytometry , growth curve, and competition experiments were independent experiments with 3 to 12 biological replicates.<br>Single-cell transcriptomics was repeated twice independently.<br>All attemps of replication were succesful.
Randomization	Biological replicates originate from randomly chosen distinct colonies selected on LB agar plates from frozen (-80°C) culture stocks. Samples were compared to the corresponding control so that no randomization process was necessary.
Blinding	Blinding for group allocation is not relevant for our study as group allocation is not part of the experimental design

Reporting for specific materials, systems and methods

We require information from authors about some types of materials, experimental systems and methods used in many studies. Here, indicate whether each material, system or method listed is relevant to your study. If you are not sure if a list item applies to your research, read the appropriate section before selecting a response.

Materials & experimental systems

n/a	Involved in the study
☒	☐ Antibodies
☒	☐ Eukaryotic cell lines
☒	☐ Palaeontology and archaeology
☒	☐ Animals and other organisms
☒	☐ Clinical data
☒	☐ Dual use research of concern
☒	☐ Plants

Methods

n/a	Involved in the study
☒	☐ ChIP-seq
☐	☒ Flow cytometry
☒	☐ MRI-based neuroimaging

Plants

Seed stocks	Not applicable
Novel plant genotypes	Not applicable
Authentication	Not applicable

Flow Cytometry

Plots

Confirm that:

☒ The axis labels state the marker and fluorochrome used (e.g. CD4-FITC).

☒ The axis scales are clearly visible. Include numbers along axes only for bottom left plot of group (a 'group' is an analysis of identical markers).

☐ All plots are contour plots with outliers or pseudocolor plots.

☒ A numerical value for number of cells or percentage (with statistics) is provided.

Methodology

Sample preparation	The promoter PtraG was cloned using HiFi Gibson Assembly into a pPROBE-NT backbone with primers traGp_Fw (5'-gttagttagggaataagccgagttttaagggagcctcgcgg-3') and traGp_Rv (5'-aggtcgactctagaggatcggccaggaagagggctaaag-3') to amplify the PtraG region from pKJK5 with overhangs to pPROBE-NT and primers MFHO38 (5'-gatcctctagagtcgacctgc-3) and MFHO37 (5'-tcggcttattccctaactaactaaag-3') for pPROBE-NT. The GFP reporter plasmids were transformed into P. putida KT2440 (negative control) and P. putida KT2440/pKJK5 following the electroporation procedure. Overnight cultures were diluted 1/50 and normalized to an OD of 0.1. These cultures (n=3) were then grown at 30°C and 250 RPM. To measure GFP intensity and abundance, samples were taken at OD0.5 and OD1.5, washed twice with PBS
Instrument	FACS Aria III, Becton Dickinson Biosciences, San Jose, CA, USA
Software	FlowJo software v10 (Tree Star Inc., USA).
Cell population abundance	Bacterial cells represented 99.6% of the events, and were identified as described in the gating strategy. A total of 50,000 bacterial cells was considered for analysis.
Gating strategy	Cell size and granulometry, were used to identify bacterial cells, using the 488 nm laser and the FSC and SSC detection channels

☐ Tick this box to confirm that a figure exemplifying the gating strategy is provided in the Supplementary Information.
